# Effect of Nanoparticles and Environmental Particles on a Cocultures Model of the Air-Blood Barrier

**DOI:** 10.1155/2013/801214

**Published:** 2012-12-23

**Authors:** Rossella Bengalli, Paride Mantecca, Marina Camatini, Maurizio Gualtieri

**Affiliations:** Department of Earth and Environmental Sciences, Research Centre Polaris, University of Milano Bicocca, Piazza della Scienza 1, 20126 Milano, Italy

## Abstract

Exposure to engineered nanoparticles (NPs) and to ambient particles (PM) has increased significantly. During the last decades the application of nano-objects to daily-life goods and the emissions produced in highly urbanized cities have considerably augmented. As a consequence, the understanding of the possible effects of NPs and PM on human respiratory system and particularly on the air-blood barrier (ABB) has become of primary interest. The crosstalk between lung epithelial cells and underlying endothelial cells is indeed essential in determining the effects of inhaled particles. Here we report the effects of metal oxides NPs (CuO and TiO_2_) and of PM on an *in vitro* model of the ABB constituted by the type II epithelial cell line (NCI-H441) and the endothelial one (HPMEC-ST1.6R). The results demonstrate that apical exposure of alveolar cells induces significant modulation of proinflammatory proteins also in endothelial cells.

## 1. Introduction

Diffusion biological barriers, such as epidermis, the gastrointestinal tract, and the respiratory epithelium, are physiologically designed to separate two different compartments in order to allow the selective passage of external substances that are essential for the organisms and to protect the body from pathogens and hazardous substances.

The alveolar region is the functional area for the gas exchange. Alveolar epithelial cells form tight and adherens junctions which play a key role in the functionality of the air-blood barrier (ABB). In addition the maintenance of the integrity of the ABB is pivotal for the physiological function of the lungs and limits the passage of inhaled material to the blood circulation.

The alveolar surface is approximately 100 m^2^ and thus is the primary target for inhaled particles and nanoparticles. Inhaled particulate interact with the apical side of the epithelial cells and might be internalised and eventually translocated to the endothelium and to the blood circulation. Moreover, even in the absence of translocation, cell-particle interactions might determine an inflammatory status that in turn facilitates particles translocation due to the loss of the selective permeability of the epithelial cells and to an increase of the permeability of the endothelial cells [1, 2]. 

Several *in vivo* studies have reported pulmonary inflammation and lung injury in response to nanoparticles (NP) exposure. Nevertheless the specific effects on the ABB are scanty due to the complexity of a precise anatomical analysis of this region of the lung. As an alternative, simpler *in vitro* models of alveolar epithelium have been widely used to study important biological and functional characteristics. However the specificity of the ABB deserves more accurate analyses possibly developing a model able to mimic the interplay of epithelial and endothelial cells. 

NPs have gained major attention in the last years due to the increase of their application in commercial products and biomedicine and the increased evidence that metal oxide particles are present in the environment as a consequence of human activities. Furthermore metal NPs have found in the last years an extensive use in industrial applications and as a consequence concern has been raised on their potential adverse effects on humans after accidental inhalation. Moreover monitoring analyses have highlighted the high presence of metal oxide NPs at sites surrounding factories, when compared to clean areas [3], and epidemiological studies have reported a correlation between the level of such NPs and the increase in pulmonary disease including exacerbation of bronchial asthma [4]. *In vitro* studies have reported cytotoxic effects for CuO NPs [5] while TiO_2_ NPs have been usually referred to as particles without significant biological effects [6]. However the results may be different depending on many factors such as the cell types used, NPs administration conditions, and the test sensitivity. Furthermore NPs may induce alveolar-capillary barrier injury causing disruption of alveolar and endothelial integrity which may determine the leakage of inflammatory mediators in the circulatory system [7, 8].

Particulate matter (PM) is considered one of the major environmental contaminants in different cities, and although great attention has been devoted to the fine fractions of PM, a main issue remains the potential effects of the coarse fraction (PM10) which has been reported to possess specific chemical and biological properties [9, 10]. In fact summer PM10 samples have been reported to be characterized by bacterial components and crustal elements, responsible for the production of proinflammatory effects.

In the present paper the effects of two metal oxide NPs (CuO and TiO_2_) and of summer PM10 sampled in Milan on a model of ABB are presented. The model consists of a human lung epithelial cell line, NCI-H441, with characteristics of both type II pneumocytes and Clara cells [11] and the human pulmonary microvascular endothelial cell line (HPMEC-ST1.6R) cultured on opposite site of a transwell filter insert [12, 13]. The ABB model has been characterized for its functional properties while particles effects have been determined by measuring the transepithelial resistance (TEER) and the release of pro-inflammatory mediators. Internalization of nanoparticles has been furthermore assessed by means of transmission electron microscopy.

## 2. Materials and Methods

### 2.1. Preparation of NPs Suspensions

nCuO (<50 nm) and nTiO_2_ (<100 nm) (Sigma Aldrich, Italy) were weighted and suspended in ultrapure sterile Milli-Q water at the concentration of 8 mg/mL. Suspensions were sonicated for 1 min with the sonicator bath Ultrasonic Soniprep 150 MSE (Sanyo) and then diluted in PBS + BSA (0,1% final concentration; Sigma Aldrich) in order to avoid particles agglomeration and optimize suspension stability. Working concentrations (0, 1, 10, 25, 50, 100 *μ*g/mL) were obtained by adding NP suspensions directly to the culture medium (OptiMEM1X, 1% FBS; M199, 1% FBS; GIBCO). NPs and NPs suspensions were characterized as reported [14]. PM10 collected and characterized as previously reported [10] has been resuspended in sterile water and added directly to the culture medium. 

### 2.2. Cells Cultures

Human pulmonary microvascular endothelial cell line HPMEC-ST1.6R was received from Dr. Ronald E. Unger (Institute of Pathology, Medical University of Mainz, Johannes Gutenberg University, Mainz, Germany) and cultured in 0,2% gelatine (Sigma Aldrich) coated flasks in M199 medium supplemented with 15% FBS (Gibco), 2 mM Glutamax I (Sigma Aldrich), 25 *μ*g/mL sodium heparin (Sigma Aldrich; Italy), 25 *μ*g/mL endothelial cell growth supplements (Sigma Aldrich), and 100 U/100 *μ*g/mL Pen/Strep (Euroclone, Italy) at 37°C, 5% CO_2_.

The human lung adenocarcinoma cell line NCI-H441 (ATCC, USA) was maintained in OptiMEM medium (Gibco) supplemented with 10% FBS and Pen/Strep (100 U/100 *μ*g/mL), at 37°C, 5% CO_2_.

### 2.3. In Vitro Alveolar-Capillary Barrier Model: Coculture of HPMEC-ST1.6R with NCI-H441

HPMEC-ST1.6R (9 × 10^4^/cm^2^) cells were placed on the lower surface of transwell filter membranes (polyester; 0,4 *μ*m pore size; Costar) coated with 0.2% gelatine and incubated for 2 h at 37°C and 5% CO_2_. The filter membranes were then turned upside down and placed in a 12-well plate filled with 1,5 mL HPMEC-ST1.6R medium (15% FBS) and incubated for 24 h.

The day after NCI-H441 (2 × 10^4^/cm^2^) cells were seeded on the top surface of the transwell filters and cultured to confluence simultaneously with the HPMEC-ST1.6R seeded on the lower surface for 11 to 13 days. To induce differentiation, the NCI-H441 cell line in cocultures was treated with 1 *μ*M Dexamethasone (Sigma Aldrich) from day 3 of cultivation. Summer PM10 (10 *μ*g/cm^2^ equivalent to 22,5 *μ*g/mL) and nCuO and nTiO_2_ suspensions (25 *μ*g/mL) were added to the apical compartment at the 11th/12th day of coculture for additional 24 h. The doses of treatment have been defined accordingly to previous results we have reported for PM10 effects [10] and by dose-response curves on the single cell lines used for setting up the air-blood barrier model (data not shown).

### 2.4. Transepithelial Electrical Resistance (TEER) Measurements

In order to determine the integrity of the *in vitro* barrier the transbilayer electrical resistance (TEER) was measured with an EVOM Volt Ohm Meter (World Precision Instruments, Berlin, Germany) equipped with a EndOhm Chamber (World Precision Instruments, Berlin, Germany). After PM and NPs exposure, before TEER measurements, transwell filters were washed with PBS and incubated with M199 medium for 10 min in order to avoid alteration in TEER values due to cellular debris and to insert handling. 

The TEER of polyester transwell filter membranes coated with gelatine 0.2%, without cells, was measured and set as blank. Barrier resistance readings (Ω∗cm^2^) were made by subtracting the resistance of the blank filter membrane and by multiplying the area of the insert (1.12 cm^2^). Resistance was reported as mean ± SE of at least three independent experiments.

### 2.5. Immunostaining

After 11-12 days of cultivation the apical compartment of coculture was washed in PBS and then fixed with paraformaldehyde (1%) for 20 min at RT. Cells were then permeabilized with Triton X-100 1%, TWEEN 1% in PBS + BSA 3% for 20 min at 4°C. The cells were then washed in PBS and incubated overnight at 4°C with rabbit anti-human *zonula occludens-1 *(ZO-1) antibody (cell signaling; 1 : 100) in PBS + BSA 1% buffer. 

Cells were then washed three times in PBS and then incubated with the secondary antibody goat anti-rabbit Alexa fluor-488 (Invitrogen Molecular Probes Srl; 1 : 1000) for 1 h. For actin staining cells were incubated 1 h with phalloidin-TRIC (Sigma Aldrich, 1 : 750). Samples were mounted on a glass slide with ProLong mount (Invitrogen Srl) and observed by an AxioScope reverted microscope (Zeiss, Germany). 

### 2.6. Cytokines Release

At the end of the exposure time cell culture media were collected and centrifuged at 1100 rpm for 5 min to remove cell debris. The final supernatants were stored at −80°C. IL-1*β*, IL-6, and IL-8 protein levels were determined by sandwich ELISA (Human Cytoset IL-1*β*, IL-6 and IL-8; Biolegend) according to the manufacturer's instructions. Absorbance was measured and quantified by a plate reader (Multiskan Ascent, Thermo Scientific Instruments) at wavelengths of 450 and 570 nm.

### 2.7. Nanoparticles Uptake

Coculture samples were prepared for transmission electron microscopy (TEM) according to the standard procedures. Briefly, cells were fixed in 2,5% glutaraldehyde for 20 min at 4°C and postfixed with 1% osmium tetroxide for 1 h, followed by dehydration in graded ethanols. Samples were then embedded in Epon/Araldite resin, and ultrathin sections were cut at the ultramicrotome (Reichert Ultracut Jung E). The sections were collected on copper grids, counterstained by lead citrate and uranyl acetate, and exanimated by Jeol JEM 1220 TEM microscope operating at 80 kV equipped with a Gatan CCD camera for digital imaging.

### 2.8. Statistical Analysis

All the experiments have been performed in independent triplicates, and data are reported as mean ± S.E. if not otherwise specified. Statistical analyses have been performed with Sigmastat 3.1 software with ANOVA test and post hoc analysis. 

## 3. Results

### 3.1. Formation of the Alveolar-Capillary Barrier

In order to estimate the integrity of the *in vitro* alveolar-capillary barrier the formation of tight junctions (TJs) was observed and the transepithelial electric resistance between cocultures measured.

**Figure 1:**
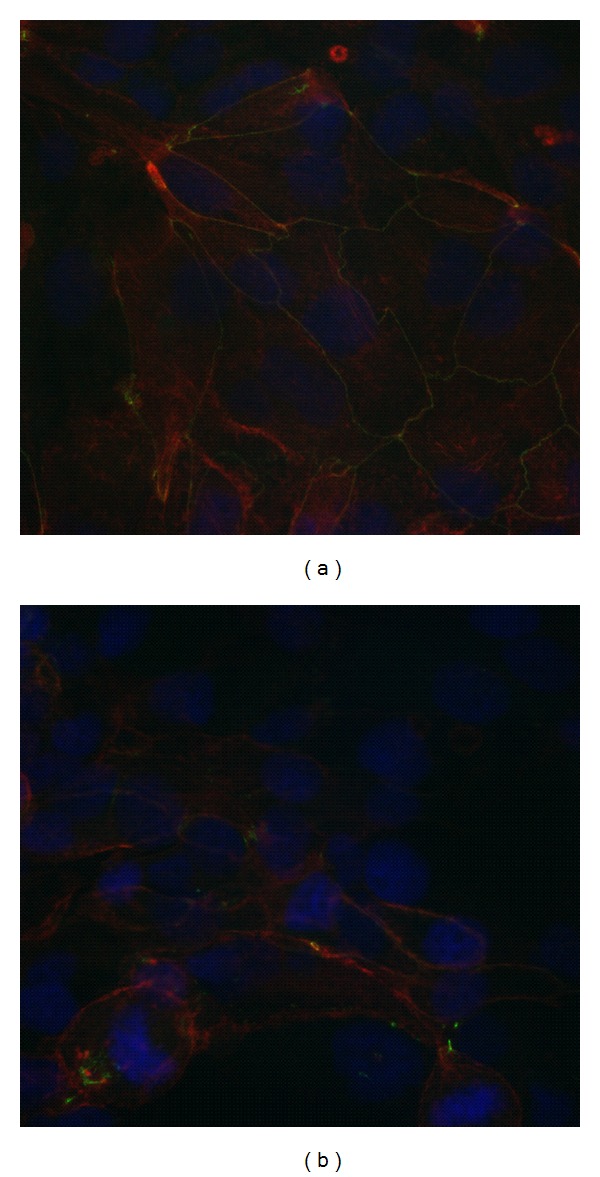
Immunofluorescent staining of the TJ cytoplasmic plaque protein ZO-1 (green) of the NCI-H441 monolayer on day 12, performed as described in Section 2. NCI-h441 differentiated with 1 *μ*M Dexamethasone after day 3 of culture were positively stained for ZO-1 at the cell-cell interface (a) confirming the formation of functional TJs while in cells without Dexamethasone treatment the ZO-1 staining clearly demonstrates the absence of TJs formation; (b) nuclei were stained with DAPI (blue), ZO-1 (green), and actin (red).

The TJs formation was assessed by the stain of the tight junctional cytoplasmic plaque protein ZO-1. Data showed that after 11–13 days of cocultures and stimulation with Dexamethazone (1 *μ*M), ZO-1 junctions were localized at the periphery of NCI-H441 cells as a continuous line delineating the limits of each cell (Figure 1(a)). The importance of the Dexamethazone stimulation is evident when comparing the ZO-1 staining of nonstimulated monolayer in which it is impossible to identify a clear membrane localization of ZO-1. 

**Figure 2:**
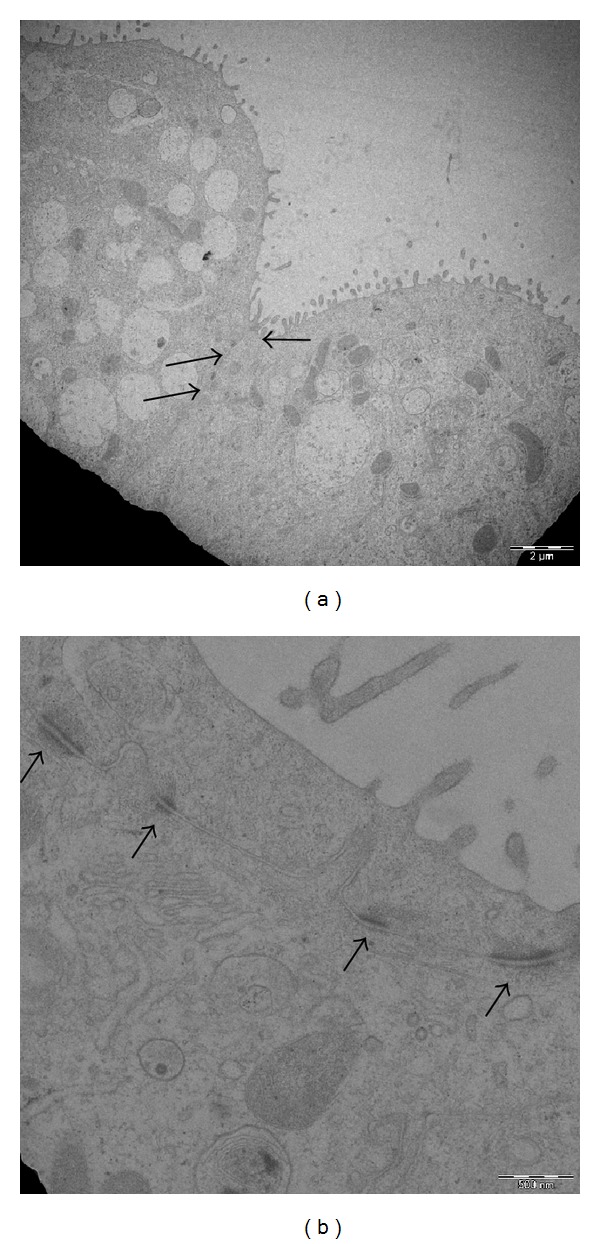
(a) and (b) Transmission electron microscope of NCI-H441 cells after 12 days of coculture. The TEM pictures show the formation of tight (↑) and adherens (*⇑*) junctions.

The formation of junctions between adjacent alveolar cells (NCI-H441) was also evident by transmission electron microscope analysis (Figure 2). 

**Figure 3:**
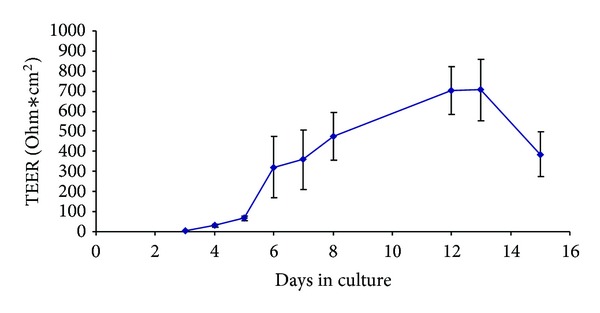
Transepithelial electric resistance (TEER) values of the ABB coculture of NCI-H441 (treated at day 3 with Dexamethasone 1 *μ*M) and HPMEC-ST1.6R. TEER values are expressed as Ω∗cm^2^. The highest values of TEER were reached after 11–13 days in culture (703 ± 118 Ω∗cm^2^). Gelatine-coated inserts without cells were used as blank. Data are expressed as means ± S.E. of seven different experiments.

**Figure 4:**
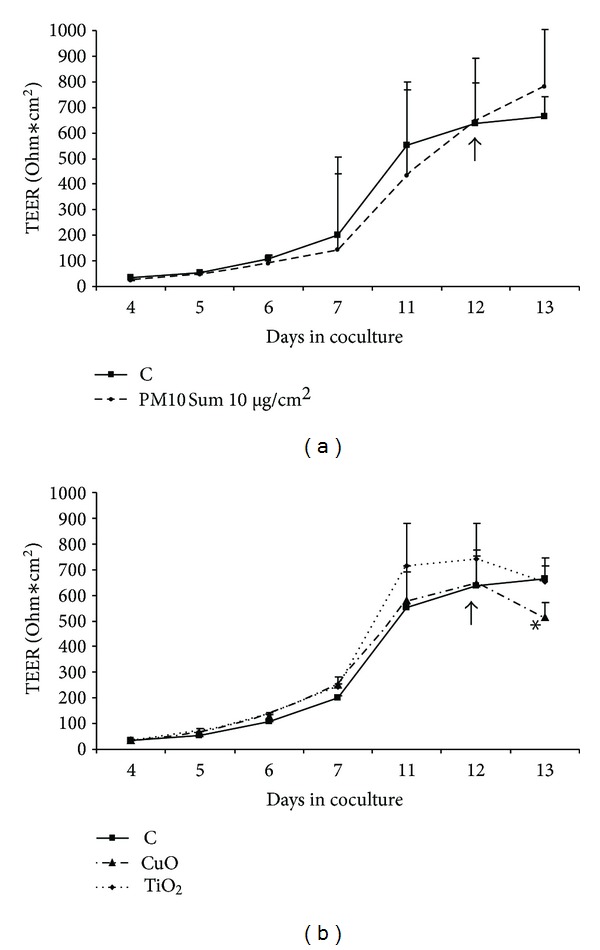
Transepithelial electric resistance (TEER) values of the ABB coculture of NCI-H441 (treated at day 3 with Dexamethasone 1 *μ*M) and HPMEC-ST1.6R. The ABB has been apically treated with PM10 (Figure 4(a)) and metal oxide NPs (Figure 4(b)). TEER values are expressed as Ω∗cm^2^ and show no significant differences between control and PM10 and TiO_2_-treated cells. Cells treated with CuO NPs showed a significant reduction in TEER values. ↑ indicates the day of apical treatment with particles. Gelatine-coated inserts without cells were used as blanks. Data are expressed as means ± SE of 3 different experiments. *Statistically different from control *P* < 0.05, ANOVA.

The integrity of the *in vitro* alveolar barrier was assessed by measuring the transepithelial electric resistance (TEER) between the cocultures. TEER was expressed as Ohm∗cm^2^, and the data showed that NCI-H441 and HPMEC-ST1.6R cocultures reached the maximum TEER at 703 ± 118 Ω∗cm^2^ (*N* = 7) after 11–13 days of cultures (Figure 3). Preliminary data showed that the treatments with PM10 (10 *μ*g/cm^2^ equivalent to 22.5 *μ*g/mL) and with nTiO_2_ (25 *μ*g/mL) did not produce significant changes in the TEER values (Figures 4(a) and 4(b)). On the contrary the TEER of the ABB treated with nCuO showed a significant reduction. The TEER value after 24 h of treatment was reduced to 514 Ω∗cm^2^compared to the control value of 665 Ω∗cm^2^ (Figure 4(b)).

### 3.2. Cocultures Exposure to nCuO, nTiO_2_, and Summer PM10: Inflammatory Response

**Figure 5:**
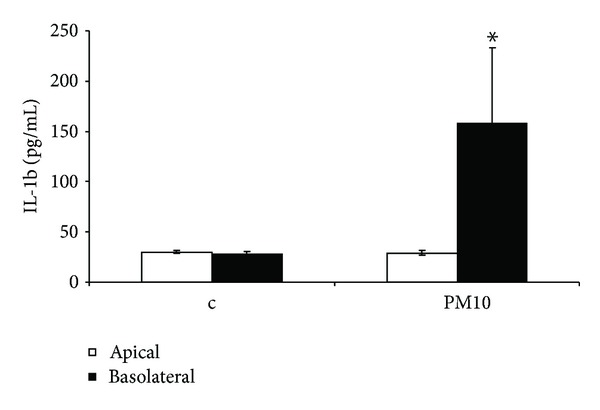
IL-1*β* release from the ABB model exposed to Milan summer PM10 for 24 h at day 12 of culture. The release of the interleukin is significantly increased in the basolateral compartment (endothelial cells) after apical exposure of the system (NCI-H441 compartment). *Statistically different from control *P* < 0.05, ANOVA.

The ABB coculture system was treated with summer PM10 and metal oxide NPs (nCuO and nTiO_2_) in order to evaluate the specific inflammatory effects produced to the ABB model. The results showed that summer PM10 induced a significant increase in IL-1*β* release from basolateral compartment (158 pg/mL versus 28 pg/mL of the control group) while the apical compartment did not produce any IL-1*β* release (Figure 5).

**Figure 6:**
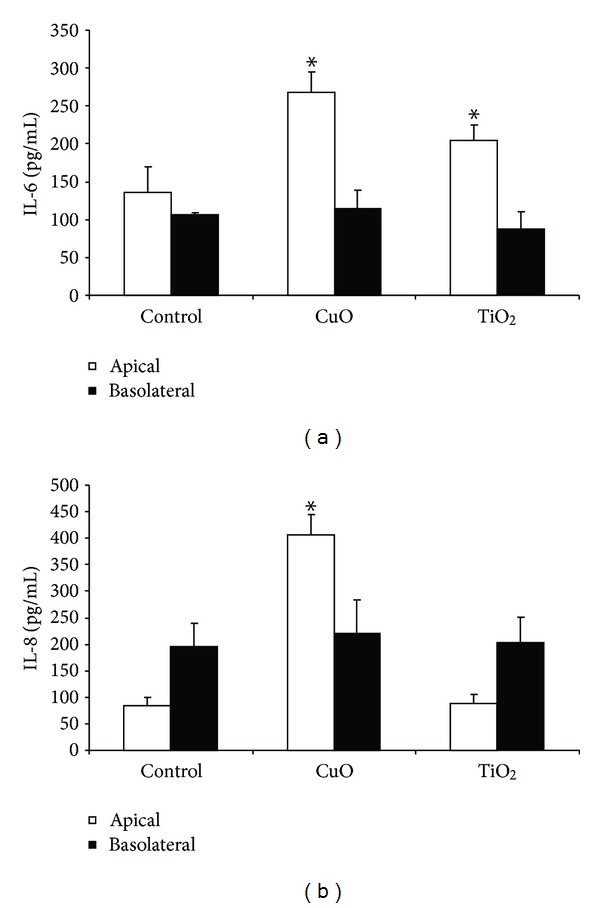
IL-6 and IL-8 release from the ABB model exposed to metal oxides NPs (25 *μ*g/mL) at day 12 of culture. The release of IL-6 is significantly increased by CuO and TiO_2_ NPs in the apical compartment (a) while IL-8 is significantly increased in the apical compartment only after treatment with CuO NPs. (b) *Statistically different from control *P* < 0.05, ANOVA.

The ABB treated with CuO showed a significant increase in IL-6 and in IL-8 release (268 pg/mL of IL-6 in treated cells supernatants versus 136 pg/m in control ones and 406 pg/mL of IL-8 in treated cells supernatants versus 83 pg/m in control ones) from the apical compartment while a significant interleukins release from the basolateral compartment was not observed (Figure 6(a)). nTiO_2_ induced an increase only in IL-6 (205 pg/mL in the treated group versus 136 pg/mL in control one) from the epithelial compartment (Figure 6(b)). NPs failed to induce significant release of IL-1*β* (data not shown).

### 3.3. NPs Internalization in Cocultures

**Figure 7:**
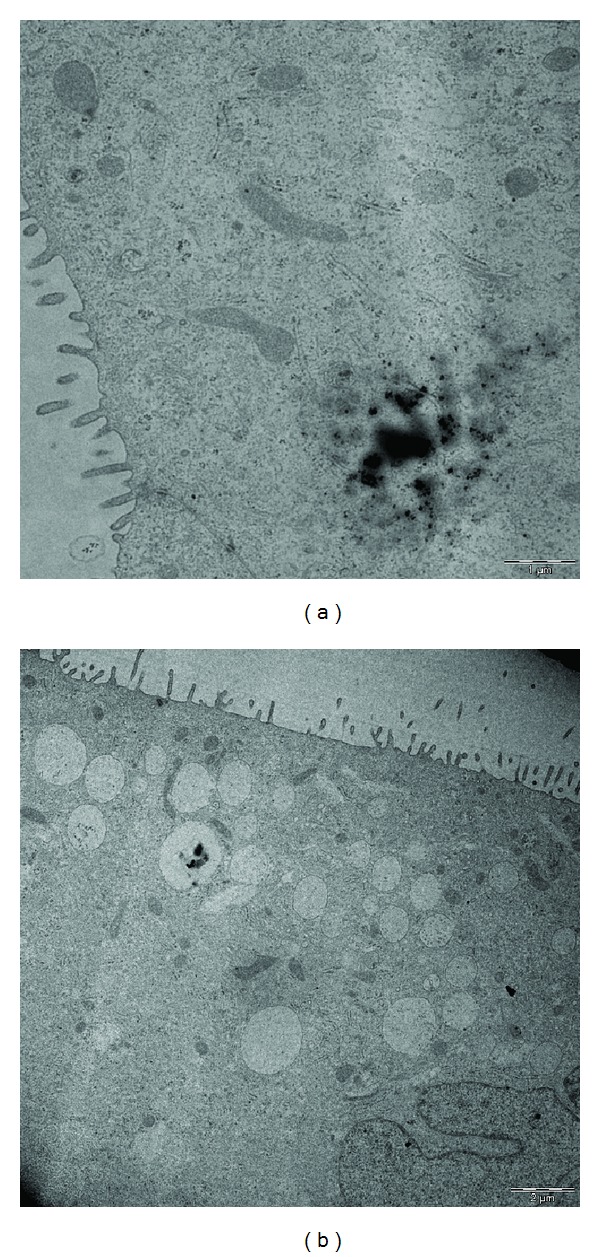
Transmission electron microscope of NCI-H441 cells after 12 days of cocultures treated with NPs. (a) NCI-H441 cells treated with CuO (25 *μ*g/mL). (b) NCI-H441 cells treated with TiO_2_ (25 *μ*g/mL). The presence of CuO NPs free in the cytoplasm can be related to the oxidative potential of these particles which determines the rupture of endosomal vesicles as well as to a different route of entry into the cells.

NCI-H441 cells of coculture experiments exposed to CuO and TiO_2_ NPs showed the internalization of CuO and TiO_2_ NPs. Interestingly nCuO was found free in the cytoplasm (Figure 7(a)) while nTiO_2_ (Figure 7(b)) was compartmentalised into vesicles.

## 4. Discussion

### 4.1. Coculture Model of *In Vitro* Air-Blood Barrier

The need of developing new models to investigate the effect mechanisms produced by inhaled toxicants has been also recently outlined [15]. The gold standard proposed for the ABB is a coculture of primary alveolar epithelial cells and human pulmonary endothelial cells [16]. Such model is expensive and subject to differences due to the primary cells availability. Therefore the development of an ABB *in vitro* system replicating the architecture and functionality of the alveolar space, based on cell lines, is of particular interest for the reduction of *in vivo* experiment too. Here we present the data obtained by the use of a model constituted of two cell types: the alveolar type II cell line, NCI-H441 and the human pulmonary microvascular endothelial immortalised cells, HPMEC-ST1.6R. NCI-H411 is a commercially available line while HPMEC-ST1.6R has been obtained by primary human pulmonary microcirculation endothelial cells (HPMEC) transfected with the plasmids pSV3neo and pC1.neo.hTERT [17]. These endothelial cells exhibited most of the characteristics of the primary human pulmonary microvascular endothelial cultured cells [13] and have been therefore selected as representative model to establish a functional ABB. 

The coculture model reached, after 11–13 days, a TEER representative of a functional barrier with a maximum of 703 ± 118 Ω∗cm^2^, in agreement with previous reports [8, 12]. Such TEER value is reached thanks to the tight junctions (TJs) and adherens junctions (AJs) usually formed between epithelial cells via highly regulated events that establish cell differentiation (apical-basolateral membrane polarity) [18]. The immunofluorescence technique, used to evidence the epithelial cells Zonula occludens-1 (ZO-1), confirmed the integrity of the ABB. ZO-1 is one of the major cytoplasmic proteins forming the TJs and has been found to be associated with the transmembrane protein occludin and to link it to the actin-based cytoskeleton [19]. After 11–13 days of cocultures and stimulation with Dexamethazone (1 *μ*M), ZO-1 junctions were clearly localized at the periphery of NCI-H441 cells as a continuous line surrounding each cell at its border. Moreover the transmission electron microscope images confirmed the formation of AJs among adjacent cells. 

The TEER of the ABB models, exposed to TiO_2_ NPs or to PM10, showed no differences with respect to the controls. These data confirm that the low doses used did not induce significant cell toxicity in the epithelial cell line. On the contrary CuO NPs induced significant effects on TEER, therefore confirming the ability of the NPs to affect the functionality of the AAB. Indeed in A549 cells the same dose of nanometric CuO have been reported to induce a 30% of cell viability reduction [14, 20]. In other cell lines, CHO and HeLa cells, CuO NPs resulted to be more toxic, and the toxicity was related to the dissolution of Cu^2+^ ions from the particles [21]. The differences, among the effects produced on the cell lines used, are likely due to different uptake processes and/or different responsiveness to the treatment. Recently it has been reported [8] that coculture systems are less sensitive to NPs-induced cytotoxicity, therefore, the low reduction of TEER here observed could be related to an overall lower responsiveness of the NCI-441 cell line when differentiated for the ABB model. Nevertheless the ability of CuO NPs to induce a TEER reduction underlines the importance of better understanding the effects of NPs on the ABB in terms of reduction of its functionality. 

### 4.2. Inflammatory Response

Inflammation processes are general responses to different lung diseases, even inflammation is a physiological response, and an uncontrolled inflammatory status may lead to adverse health effects. The ability of inhaled particles to induce the release of proinflammatory mediators has been largely used as marker of potential toxic effects [22, 23]. However the data available refer to monoculture of lung epithelial cells and the few data exist on lung endothelial cells. 

The biochemical crosstalks between the cell types constituting the ABB are fundamental in maintaining the barrier homeostasis [24–26].

The NPs ability to induce an acute inflammatory response in lungs of rodents has been reported [27, 28]. *In vitro* monocultures of both lung epithelial and endothelial cells have been used to define the proinflammatory potential of metal oxide NPs [23, 29–31]. Nevertheless the data on significant model of ABB are rather scanty [8]. In agreement with our previous results [14] the ability of CuO and TiO_2_ NPs to induce a significant inflammatory response in the apical side of the ABB mode is shown. CuO NPs induced a significant release of the proinflammatory mediators, IL-6 and IL-8, and TiO_2_ increased only the release of IL-6 while both the NPs did not induce significant release of IL-1*β*. The lack of response of the basolateral side can be related to the integrity of the ABB which prevents the passage of NPs and/or ions towards the endothelial compartment although additional data are needed to elucidate the basolateral responses. However, Papritz et al. [32] showed for Cd^2+^ ions that, after apical exposure, the basolateral response was significant at concentrations which determined a decrease in TEER values. 

Interestingly the apical treatment of the ABB with PM10 induced a basolateral release of IL-1*β*. This result is in accordance with others [8, 16] which obtained a basolateral response of endothelial cells after treatment of the epithelial cells at the apical side. IL-1*β* release confirms the biochemical crosstalk between the two sides of the membrane and supports the functionality and representativeness of our ABB model. Further studies will be needed to understand the mechanism by which PM10 induces IL-1*β*. Preliminary results obtained in our lab on THP-1-derived macrophages suggest that the inflammasome activation plays a central role (data not shown). The ability of PM in triggering the release of a potent inflammatory mediator such as IL-1*β* has to be carefully taken into account. Van Eeden et al. [33] showed a significant increase of circulating IL-1*β* in population exposed to high level of PM10; furthermore increased expression of IL-1*β* has been related to lung injury and lung tissue remodelling [34]. Our ABB model thus furnishes a suitable system to be used for studying lung damage mechanisms and systemic inflammation following exposure to environmental and engineered NPs. 

### 4.3. NPs Internalization in Cocultures

Several *in vivo* and *in vitro *studies showed the translocation of NPs through the respiratory epithelium and the distribution of NPs in secondary organs such as liver, kidney, and brain [35]. Nevertheless there are still controversial evidences about NPs translocation through the alveolar barrier. ABB *in vitro* models can thus furnish a helpful system for investigating the actual translocation process across the barrier. Intake of submicrometric and nanometric particles in culture of epithelial cell has been widely reported [36–38]. Our data showed that both nCuO and nTiO_2_ were found internalised in epithalial cells; however while nTiO_2_ particles were found as agglomerates in cytosolic vesicles, suggesting an endocytosis-mediated mechanism of internalization, nCuO particles were found as free aggregates in the cytoplasm [14, 39]. nCuO were found as free aggregates into the cytoplasm. This difference might be related to the higher oxidative potential of CuO NPs in comparison with TiO_2_ ones, and this ability may lead to a lipid peroxidation in the endosome vesicles, with a consequent presence of free CuO NPs in the cytoplasm. 

However after 24 h of treatment, NPs were not found internalised in the basolateral compartment of endothelial cells. The inability of nCuO and nTiO_2_ to cross the membrane filter pores of 0.4 *μ*m can be due to the tendency of NPs to form aggregates that are physically unable to pass through the membrane pores. Indeed it has been proposed that, even without a cell layer on the top of the filter, NPs which tend to agglomerate are not able to cross the membrane [40]. The same authors have demonstrated the capability of certain NPs to cross an *in vitro* model of ABB, in experiments that used a membrane filter with 3 *μ*m pore size and A549 cell line, which were not able to form TJs. 

Our data thus suggest that in presence of a functional ABB, the possibility of NPs to cross the barrier is rather scanty, although additional data are needed. For example, the use of air-liquid interface treatments, which drastically reduce the NPs agglomeration tendency in culture media, will be of particular interest in defining the translocation ability of nano objects. 

In conclusion the ABB model reported here is a suitable system for studying the toxicological events at the alveolar level. Our date demonstrate that the two cell types are able to cross communicate, as evidenced by the IL-1*β* release in PM10-treated samples, although the passage of particles seems not to occur. Further studies will help to understand which molecules are responsible for the cells crosstalk and if, under different cell treatment conditions, the NPs translocation is really possible in a functional ABB.
